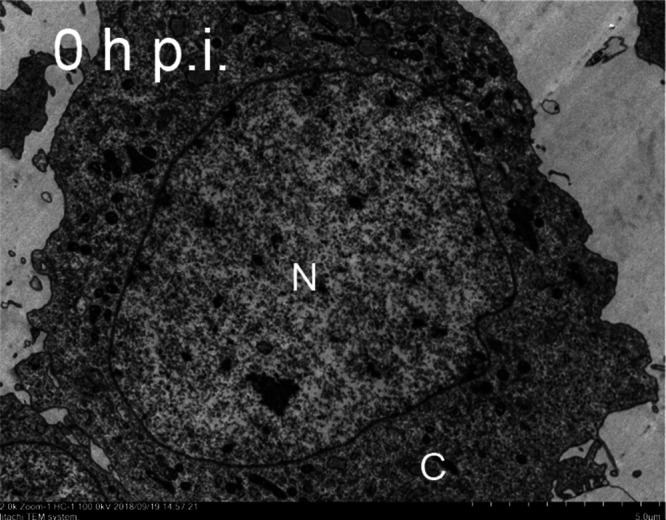# Erratum for Dong et al., “Nosema bombycis microRNA-like RNA 8 (Nb-milR8) Increases Fungal Pathogenicity by Modulating *BmPEX16* Gene Expression in Its Host, Bombyx mori”

**DOI:** 10.1128/spectrum.04342-22

**Published:** 2022-12-08

**Authors:** Zhanqi Dong, Ning Zheng, Congwu Hu, Boyuan Deng, Wenxuan Fang, Qin Wu, Peng Chen, Xuhua Huang, Na Gao, Cheng Lu, Minhui Pan

## ERRATUM

Volume 9, no. 2, e01048-21, 2021, https://doi.org/10.1128/Spectrum.01048-21. Page 3, Fig. 1A: The wrong version of the control (0 h p.i.) image was uploaded during preparation of final files for publication. The correct 0-h p.i. panel is shown here (Fig. 1). We apologize for this error and state that this does not change the scientific conclusions of the article in any way.[Fig fig1]

**Figure fig1:**